# The Effect of Needle Type, Duration of Surgery and Position of the Patient on the Risk of Transient Neurologic Symptoms

**DOI:** 10.5812/aapm.6916

**Published:** 2013-03-26

**Authors:** Farhad Etezadi, Kourosh Karimi Yarandi, Aylar Ahangary, Hajar Shokri, Farsad Imani, Saeid Safari, Mohammad Reza Khajavi

**Affiliations:** 1 Department of Anesthesiology, Sina Hospital, Tehran University of Medical Sciences, Tehran, Iran; 2 Department of Neurosurgery, Tehran University of Medical Sciences, Tehran, Iran; 3 Department of Anesthesiology, Rasoul Akram Medical Center, Iran University of Medical Sciences (IUMS), Tehran, Iran

**Keywords:** Neurologic Manifestations, Anesthetics, Local

## Abstract

**Background:**

The incidence of transient neurologic symptoms (TNS) after spinal anesthesia with lidocaine is reported as high as 40%.

**Objectives:**

This prospective clinical trial was designed to determine the incidence of TNS in patients who underwent spinal anesthesia with two different needles, in two different surgical positions.

**Patients and Methods:**

The present randomized clinical trial was conducted on 250 patients (ASA I-II), who were candidates for surgery in supine or lithotomy positions. According to the needle type (Sprotte or Quincke) and local anesthetics (lidocaine and bupivacaine) all patients were randomly divided into four groups. After performing spinal anesthesia in sitting position, the position was changed into supine or lithotomy, according to surgical procedure. The patients were observed for complications of spinal anesthesia during the first five post-operative days. The primary end-point for this trial was to recognize the incidence of TNS among the four groups. Our secondary objective was to evaluate the effect of patient's position, needle type, and duration of surgery on the development of TNS following spinal anesthesia.

**Results:**

TNS was most commonly observed when lidocaine was used as anesthetic drug (P = 0.003). The impact of needle type, was not significant (P = 0.7). According to multivariate analysis, the duration of surgery was significantly lower in cases suffering from TNS (P = 0.04). Also, the risk of TNS increased following surgeries performed in lithotomy position (P = 0.00).

**Conclusions:**

According to the results of this clinical study, spinal anesthesia with lidocaine, and the lithotomy position in surgery increased the risk of TNS.

## 1. Background

Following spinal anesthesia, some neurologic complications are expected to occur. Low back pain, radiating to the buttock and lower extremities, is the major symptom of such neurologic complications. The pain is mostly transient and usually fades within 24-48 hours. In 1993, Schneider et al. (1) published a case report purportedly describing the first cases of this clinical manifestation which is currently known as transient neurologic syndrome (TNS) (2, 3). Among local anesthetics used in spinal anesthesia, lidocaine is a commonly used agent. However, very high risk grading for development of TNS is also attributed to this drug. The most significant risk factors leading to these symptoms are not well known. Few studies have investigated the effect of the patients' position combined with the needle type.

## 2. Objectives

In the present study, we evaluated the incidence of TNS among the four groups according to the type of anesthetic agents and needles. We also evaluated the effect of patient's position and duration of surgery on the development of TNS following spinal anesthesia.

## 3. Patients and Methods

A double- blind randomized clinical trial (RCT) was designed to evaluate 250 candidates of elective surgeries from January 2011 to December 2011. The approval of the Ethics Committee of Tehran University of Medical Sciences was obtained and randomization was achieved by a computer-generated block of numbers and sealed envelope technique. All patients were between 18 to 60 years old and their ASA score was I or II. Subjects suffering from neuromuscular disease, spinal canal stenosis or vertebral disc disease, femur fracture or the fracture of pelvis, diabetes mellitus, morbid obesity, and those having history of neurologic complications following spinal anesthesia were excluded from the study. Various stages of study were explained meticulously to all patients and written consent was obtained from them .All cases were randomly divided into four groups according to the needle type (25 gauge Sprotte or Quincke) and local anesthetics (lidocaine or bupivacaine). In the first and second groups, 1.5-2mL from 5% hyperbaric lidocaine (Orion Pharma) was used for spinal anesthesia, and the third and fourth groups received 2.5-3mL from 0.5% isobaric bupivacaine (MYLAN) for the same purpose. Based on described style and through Sprotte needle, 62 patients received lidocaine, and another 62 received bupivacaine. Through Quincke needle, bupivacaine was injected in 63 patients and lidocaine in the other group of 63 cases. After EKG monitoring, non-invasive blood pressure measurement, pulse oximetry, and infusion of 8 mL/kg from normal saline, spinal anesthesia was performed in sitting position at L2-L3 or L3-L4 levels by the same anesthesiologist. The dose of anesthetic drug was tailored by the height of patients. Intra-operative hypotension (MAP reduction more than 20% of the baseline) was treated with injection of 5-10 mg from ephedrine and infusion of 200 mL from Ringer's solution. Bradycardia (heart rate < 50  bpm) was treated with 0.5 increments of atropine, and hypotension (systolic BP < 90  mmHg) with 5  mg increments of ephedrine. According to the type of surgery, the operation was performed in the supine or lithotomy positions. All cases were ambulatory within the first post-operative day. The potential neurologic complications were monitored every eight hours within the first two post-operative days, and every 24 hours for the following three days. During each post-operative control, the patients were asked to report any of the following symptoms, which were considered as the criteria for TNS development: any type of bilateral or unilateral pain, numbness, or hyperalgesia in the back; or radiating pain to waist, buttock, hip, or anterior or posterior regions of leg or thigh. The severity of pain was assessed by Visual Analogue Scale (VAS). All patients were remained hospitalized for at least 48 hours after the operation. The symptoms of TNS and any other complications were observed by a neurosurgeon that was blinded to surgical position of patients and the type of drugs and needles used for spinal anesthesia. Additional investigations for whom complaining from TNS, such as MRI and infection workup, were only performed when neurologic deficit or any sign of infection was discovered by the neurosurgeon through his detailed physical examination to rule out other etiologies. In patients with VAS score more than three, administration of pethidine and NSAID was considered. After discharge, the patients were visited for at least three consecutive days and any symptoms of TNS were evaluated. The sample size of the study was calculated according to the report of Hampl et al. (2) on the incidence of TNS development after spinal anesthesia with lidocaine. To detect a difference between the treatments, at the 95% level of significance (α = 0.05), with 80% power (β = 0.2), and assuming at least 20% change in the incidence, approximately 60 patients were required for each of the four groups. The Cox proportional hazard model was used for multivariate analysis and SPSS 11 was used for statistical analysis. The incidence of TNS development among the groups was analyzed with X2 test and group data were expressed as mean ± SD numbers and percentages. Differences were considered significant for P values less than 0.05.

## 4. Results

250 patients with mean age of 46.4 ± 15, consisting of 45 women and 205 men, were included in this study. Demographic characteristics and important risk factors, especially position of the patients during the surgery, age, sex, and duration of procedure, were not significantly different between the four groups (Table 1). Type of surgery, position of the patients during surgical procedure, and duration of surgery are shown in Table 2. 99 cases (39.6%) reported symptoms of TNS in the post-operative visits. Table 3 demonstrates the incidence of TNS according to the type of local anesthetic and needle type, and Table 4 demonstrates the incidence of TNS in four groups of the study. 77 patients complained from lumbosacral pain (with radiation to the lower extremity and aggravation in the sitting position). In the remaining 22 cases, the pain was sharp and severe, mostly located in the thigh. Most of the patients reported a pain with the measure of six or seven according to VAS scaling system. VAS scores of patients who suffered from TNS were compared between the groups and no significant difference was found (P = 0.25). Out of 125 patients anesthetized with lidocaine, 85 patients suffered from low back pain, and neurogenic pain in the buttock or thigh. In the majority of these cases the symptoms were severe enough to justify analgesic consumption. The pain was often significantly diminished at the time of discharge. Nevertheless, 12 cases suffered from prolonged pain for which prescription of NSAIDs was required after the discharge. The average length of surgery was significantly less in the patients suffering from TNS (53 ± 12 minutes) compared to whom without post-operative TNS (69 ± 10 minutes). Most of the patients with post-operative TNS received lidocaine and underwent surgery in the lithotomy position. The combination of these factors (i.e. lidocaine and lithotomy position) significantly increased the risk of this complication (P = 0.002) compared to combination of bupivacaine and supine position factors. Table 5 shows multivariate analysis of different influencing factors.

**Table 1:** Demographic Characteristics and Important Risk Factors among The Four Groups

	Group 1: Lidocaine + Sprotte	Group 2: Lidocaine + Quincke	Group 3: Bupivacaine + Sprotte	Group 4: Bupivacaine + Quincke
Age, y	47.1	46.0	46.2	46.3
Sex, male/female	49/13	51/12	51/11	54/9
Duration of surgery, min	64.1	64.2	62.1	59.6
Position, supine/lithotomy	33/29	33/30	35/27	37/26
Type of surgery				
Varicocele	11	11	12	13
TUL and TUR	29	30	27	26
Herniorrhaphy	8	9	9	9
Surgical fixation of lower extremity fracture ^a^	14	13	14	15

Abbreviations: TUL, transurethral lithotripsy; TUR, Transurethral resection of the prostate.

^a^Only the cases with fractures below the knee were included.

**Table 2:** Type of Surgery and Patients’ Position

Type of Surgery/Position	Supine	Lithotomy	Total	Duration of Surgery, Minutes
**Varicocele **	47	-	47	60 ± 11
**TUL and TUR **	-	112	112	45 ± 18
**Herniorrhaphy**	35	-	35	70 ± 12
**Surgical fixation of lower extremity fracture **	56	-	56	95 ± 17

Abbreviations: TUL, transurethral lithotripsy; TUR, transurethral resection of the prostate.

**Table 3:** The Incidence of Transient Neurologic Symptoms (TNS) According to the Drug and the Type of Needle Used

Complication	Lidocaine	Bupivacaine	Quincke	Sprotte	Total
**TNS, No. (%)**	85 (68)	14 (11)	58 (46)	41 (33)	99 (39.6)
**P value**	0.003	0.003	0.7	0.7	

**Table 4:** The Incidence of TNS in the Four Groups of the Study

Incidence/Group	Group 1: Lidocaine + Sprotte	Group 3: Lidocaine + Quincke	Group 2: Bupivacaine + Sprotte	Group 4: Bupivacaine + Quincke
**TNS, No. (%)**	35 (56)	50 (79)	6 (9)	8 (13)

**Table 5:** Multivariate Analysis of Different Influencing Factors

	Relative Risk (95% CI)	P value
**Agent: Lidocaine vs. Bupivacaine**	6.18 (4.7-7.9)	0.003
**Needle: Quincke vs. Sprotte**	1.39 (0.7-1.8)	0.7
**Position: Lithotomy vs. Supine**	4.1 (3.0-4.9)	0.008
**Duration of surgery: less than one hour vs. more than one hour**	1.63 (1.1-2.3)	0.04

## 5. Discussion

Several factors influenced on the development of low back pain and neurologic complications following spinal anesthesia. The type of drug used for anesthesia was a particularly important factor in this regard, especially when the injected agent was lidocaine. Risk of neurologic complications following the injection of other drugs has been reported to be less than that of lidocaine (4, 5). In this study, the incidence of adverse neurologic symptoms with lidocaine was significantly more compared to those with bupivacaine. This finding was consistent with previous reports in the literature (6). 39.6% of the total cases anesthetized with lidocaine or bupivacaine suffered from TNS in this study. This incidence was high, when compared with reported range in the literature. According to previous reports, neurologic symptoms developed in 10-40% of the patients following spinal anesthesia with lidocaine (7, 8). A significant number of our patients underwent surgery in the lithotomy position, which may be responsible for such inconsistency. Different solutions of lidocaine (1%, 2%, and 5%) were used for spinal anesthesia and all were reported to induce TNS (9). The exact mechanism of this phenomenon was not clarified. For the first time in 1993, Schneider et al. reported TNS development following spinal anesthesia (1). Following development of post-anesthetic pain in the buttock with radiation to lower extremities in four patients anesthetized with 5% lidocaine and 7.5% dextrose, they suggested that high osmolarity of lidocaine may be responsible for these symptoms, but further investigations using different concentrations with various osmolarities, failed to show any significant effect on the risk of TNS (9) According to our results, patients' position during surgery had a significant influence on the incidence of TNS. Out of total 112 operations performed in lithotomy position, 82 cases suffered from low back pain and paresthesia in lower extremities, whereas the rate of low back pain following surgeries performed in supine position was very low. Surgical position was still an independent risk factor for development of TNS after multivariate analysis. The normal spinal lordosis was maintained throughout the procedures performed in supine position, while the lumbar lordosis decreased in lithotomy position and lumbar part of spinal column was straightened during the operation. Muscles, tendons, joints, and nerve roots of cauda equina were significantly stretched in this position. This, subsequently, increased the risk of low back pain in post-operative period (10, 11). In fact, it seems that TNS development is multi-factorial and other parameters, other than the type of injected drug for spinal anesthesia, can aggravate or produce these symptoms. No significant difference could be discovered between the cases in regard to the type of needle used for spinal anesthesia. Identical drug distribution in the subarachnoid space was reported for both needles (12). Quincke needles caused more dural trauma compared to Sprotte needles. Contrary to the previously reported effect of dural trauma on the incidence of post-dural puncture headache, the intensity of dural injury did not seem to be a significant influencing factor on development of TNS following spinal anesthesia .According to our data; prolonged duration of surgery did not increase the risk of TNS development. In fact, duration of surgery was even significantly lower in cases suffering from TNS. It should be added that since P value after multivariate analysis was 0.044 (close to 0.05) and this finding was not consistent with the results of previous reports, we preferred to interpret it with caution. Obviously, more studies are required to assess the relationship between the duration of surgery and TNS development. Spinal anesthesia with lidocaine involved more risk for post-operative TNS development compared to that with bupivacaine. Also, the risk was further increased in the lithotomy position. The incidence of TNS was not significantly different with regard to the type of needle used for anesthesia.
